# The relevance of different trust models for representation in patient organizations: conceptual considerations

**DOI:** 10.1186/s12913-017-2368-z

**Published:** 2017-07-11

**Authors:** Helene Gerhards, Karin Jongsma, Silke Schicktanz

**Affiliations:** 1 Institute of Political Science, University of Duisburg-Essen, Lotharstraße 65, 47057 Duisburg, Germany; 2 Department of Medical Ethics and History of Medicine, University Medical Centre Göttingen, Humboldtallee 36, 37073 Göttingen, Germany

**Keywords:** Collective decision-making, Health care, Patient Organization, Patient participation, Representation, Germany

## Abstract

**Background:**

Trust within organizations is important for ensuring members’ acceptance of the organization’s activities and to expand their scope of action. Remarkably, Patient Organizations (POs) that often both function as a forum for self-help and represent patients on the health-political level, have been understudied in this respect. This paper analyzes the relation between trust and representation in POs. We distinguish between two models of representation originating from political theory: the trustee and delegate model and between two types of trust: horizontal and vertical trust.

**Methods:**

Our theoretical approach is illustrated with an analysis of 13 interviews with representatives of German POs.

**Results:**

We have found that the delegate model requires horizontal trust and the trustee model vertical trust. Both models: horizontal/delegate and vertical/trustee exist within single POs.

**Conclusions:**

The representation process within POs demands a balancing act between inclusion of affected persons and strategically aggregating a clear-cut political claim. Trust plays in that process of coming from individual wishes to collective and political standpoints a major role both in terms of horizontal as well as vertical trust. Horizontal trust serves the communication between affected members, and vertical trust allows representatives to be decisive.

## Background

That trust plays an important role in organizations is a fact recognized and studied by several scholars e.g. Luhmann [1]. Remarkably, Patient Organizations (POs) have been understudied in this respect. POs are particularly interesting organizations to investigate with regard to trust and representation, because they negotiate about health care access and provision, and are often also a kind of health provider themselves, by offering self-help groups and even supplemental care [2]. POs are sometimes criticized with regard to their (lack of) knowledge for influencing complex health policy decisions [3] or risk of instrumentalization by the pharma industry [4]. Nevertheless, the idea that patients should have a say in health policy is widely accepted on the grounds that they are the ones directly affected by these decisions [5]. Important questions concerning the means and legitimate ways of representing patients’ needs and interests are however rarely addressed [6].

One important presumption is that POs play an important role in health care decision-making and represent the patients’ interests in the political arena [5, 7–9]. The idea of establishing POs has its roots in the health social movement of the 1970s in Europe and North America [7]. By then, patients had begun to question hierarchical power structures in the health care system as well as in medical practice and research. As a result, patient movements, including POs, are traditionally neither highly integrated nor strongly organized, but have to be inclusive and are mostly defined by their purpose of emancipated ‘voicing’ [8]. Nevertheless, over the last couple of years, many POs have professionalized as public and political actors. This professionalization means that they have evolved as recognized stakeholders in policy making, are steadily using political lobbying channels and have (semi) professional spokespersons [10]. Representatives of these collectives are often elected or nominated, but are sometimes also self-declared [11]. This points to the critical relationship of internal decision-making processes, trust and representative structures *within* these organizations.

The link between trust and representation builds on theoretical discussions on representation: Melissa Williams [12] and Nadja Urbinati [13] have stated that fair representation requires relationships of trust between individuals and representatives based on shared experiences, perspectives, and interests. Furthermore, trust within organizations is of utmost importance for ensuring members’ acceptance of the organization’s actions and activities and to expand their scope of action [14].

In the present work, we examine the conceptual relationships between different types of trust and different representation styles in POs and illustrate how these conceptual models work in practice by analyzing these relationships in empirical material.

By theoretically differentiating between two styles of representation (delegate and trustee) and two concepts of trust (horizontal and vertical) in organizations, we will distinguish particular ways in which representatives can give voice to affected people in political discourses and decision-making. Drawing on interviews with different PO representatives, we will analyze the ways that representatives themselves understand their political role^1^ (delegate/trustee) and which role different types of trust (horizontal/vertical) play for representation in POs. Furthermore, we discuss the potentials and problems of different representation-trust relationships. Overall, we aim to contribute to a better practical understanding of internal structures of POs and to a more nuanced conceptual framework for the relation between trust and representation in health care organizations such as POs.

### Theoretical considerations of the two key concepts: representation and trust

#### Representation

The topic of proper representation in a pluralist society and democratic political system, as well as the normative expectations with regard to representation has been and remains an important issue in political theory and practice [15]. As social movements scholars saw value in the function of civil society organizations (CSOs) for purposes of political representation, also representation processes in and by political organizations are increasingly studied [16–18]. POs bring claims to the fore in public and political arenas based on the perspective of patients [17] and are in this respect CSOs where representation practices take place. Representation research answers the question which conditions must be met so that representatives of civil society organizations can reflect the stakeholder group’s claim in the political realm in a legitimate way:

“It is of fundamental relevance that an association structures itself in a way that fosters a series of interactional loci, so as to increase communicative flows. Associations must guarantee the existence of several spheres of interlocution, which enable a permanent encounter and confrontation of discourses and ideas. This is the only way, an association may show its *plurality* and its *adjusting capacity*, which are essential attributes for the exercise of effective representation. A representative must be in permanent metamorphosis so as to reconstruct its bonds with the represented.” [19] 127, original emphasis, see also [20].

Eulau et al. [21] introduced the distinction between the focus and style of representation: Focus refers to the group represented (constituency voters, party voters or the nation). The constituency of POs can refer to patients affected by a single disease (single-disease POs) or include people with a variety of conditions united in an umbrella organization. The representation style refers to the manner in which representatives fulfill their role. This role is problematized by Hannah F. Pitkin as follows:

“The question at issue may be summarized as: Should (must) a representative do what his constituency wants, and be bound by mandates of instructions from them; or should (must) he be free to as to act as seems best to him in pursuit of their favor?” [22].

This question is found in two different concepts of representation in classical political theories. John Stuart Mill acknowledges the importance of culture and morality of representation and the possibility to create responsive representation along a delegation principle:

“For, let the system of representation be what it may, it will be converted into one of mere delegation if the electors so choose. [ … ] By refusing to elect any one who will not pledge himself to all their opinions, and even, if they please, to consult with them before voting on any important subject not foreseen, they can reduce their representative to their mere mouthpiece, or compel him in honour, when no longer willing to act in that capacity, to resign his seat.” [23].

The other alternative referring to a rather autonomous representative is preferred by conservative political thinker Edmund Burke:

“Your representative owes you, not his industry only, but his judgment; and he betrays, instead of serving you, if he sacrifices it to your opinion. (…) To deliver an opinion, is the right of all men; that of constituents is a weighty and respectable opinion, which a representative ought always to rejoice to hear; and which he ought always most seriously to consider. But *authoritative* instructions; *mandates* issued, which the member is bound blindly and implicitly to obey, to vote, and to argue for, though contrary to the clearest conviction of his judgment and conscience,-- these are things utterly unknown to the laws of this land, and which arise from a fundamental mistake of the whole order and tenor of our constitution.” [24], emphasis in original text.

In current political theory these two main models are still relevant. The first approach is commonly described as the ‘delegate model’ of representation [25], the second is referred to as ‘trustee model’ of representation [26]. The delegate model holds that representatives are bound closely to the mandate of their stakeholders [27]. Often a majority vote or a very extensive process of deliberation between the members of an interest group precedes the channeling of the members’ shared perspective and common claims into the political system. It would be too narrow to describe the representative in this constellation merely as messenger because this would implicate a de-politicization of the representative’s role [28]. In this conception greater emphasis lies on the inclusion and consideration of arguments of those who will be affected by allocation conflicts and political decision-making outcomes.

The second model, the ‘trustee model’, would allow the (elected) spokesperson of a PO to adhere to the rules and conditions of the relevant political arenas in order to achieve the best result for the represented in a political conflict. In the trustee model, representatives are assigned to have political ‘wisdom’: professional experience and knowledge to help them to achieve a good outcome for their constituency [22].

One may have noticed that ‘trustee’ contains the word ‘trust’. One may therefore assume that trust plays a special role for trustee representatives. There are however several conceptualizations of trust– it would therefore be a too hasty conclusion to argue that trust is only required for trustee representation. In the following we will describe two concepts of trust.

### Trust

Trust is one of the most extensively examined concepts in social science – including how to understand trust between individuals or between individuals and (social, political, organizational) institutions, what the difference is between trust, trustworthiness and distrust and why trust is needed see e.g. [29].

In the political realm, one can roughly differentiate between two main forms of trust. On the one hand, trust is essential for the representative’s political capital – (professional) representatives must be trusted in their intentions *towards* the represented, their strategic work and their competent judgment in political bargaining [30]. Trustworthiness is, in this understanding, a capacity to commit oneself to fulfilling the legitimate expectation of others on the grounds of election and professional expertise [31]. David Easton stresses the role of generalized attitudes like trust in and support of representatives, which he sees as being based on social capital or a “reserve of goodwill” [32]. He argues that a representative accumulates trust through qualities, which can point to competence like efficient performance and (previous) success. To coin a name for this type of relationship of political trust we suggest *vertical type of political trust* referring to the constellation between the body of the constituency and the political functionary who acts autonomously on their behalf. Trust can be understood as the credit granted to the representative in his/her political role – that the person is not only competent but also acts in the constituency’s best interest [33].

Trust can also be built deliberatively *between* people who confide thoughts and feelings to each other, coping with the same challenges and problems experiencing severe life circumstances [34]. Then, trust takes an effect as an opener and promoter of communication among those who share similar experiences or the same social or political concerns. This understanding of trust emerges from a horizontal (mediated) interaction between the group members respectively, so we coin the term *horizontal type of political trust*. Following this conception, trust is both a mean and result of socialization that binds members of a group to one another and to the collective as a ‘symbolic body’ made up of affected people [35] – for example the collective of those who live with a handicap or a chronic disease. In pluralistic and democratic societies close ties exist between the cultivation of trust and the emergence of solidarity [36], that are important for democratic policy and voicing [37]. Horizontal trust is related to processual communicative acts and procedures of collective assurance to reduce uncertainties about dissent regarding interest or shared values.

## Methods

The empirical part of our study consists of an in-depth secondary analysis of interviews with German PO representatives (Table 1). Thirteen interviews with representatives of four different POs for specific conditions and three representatives of the German Federal Joint Committee (in German: Gemeinsamer Bundesausschuss, G-BA)^2^ were selected (Table 2) from a larger sample of 15 semi-structured interviews with patients and representatives. We excluded interviews with members without any representative role, as these were not relevant for the questions regarding self-assessed representation styles. The original focus of these interviews was on the relation between autonomy and trust.^3^

**Table 1:** Overview of our sample

Organization	*N* =	Interviewee affected	Year established	Organizational structure & membership composition	Membership size	Self-claimed Goals
German Society for Muscle Disease	2	No/No	1965	Board of directors (3-9 members); scientific advisory board (5 members); volunteers; family members and patients	ca. 8000	To offer consultancy; to support research and public relations to make the disease known to the public; engaged in health policy for representation of interests
Association for People with Locked- In-Syndrome Self- help and Friends	2	No/No	2000	Board of directors (3 members); advisory board; family members and patients	Ca. 150	To ameliorate and facilitate therapy for patients; conducting educational work for patients and their family members, therapists and doctors; to create publications and establishing a documentation center; organize international meeting with experts and affected persons
German Alzheimer Society - Dementia Self-Help	3	No/No/Y es	1989	Board of directors; advisory board with people with dementia is convened; family members and patients	Ca. 15.000	To increase understanding and support of the public towards people with dementia via public relations work; to improve possibilities to learn how to live with dementia, and self-management of relatives; organizing seminars with local and regional groups; to offer consultancy; to support scientific research and develop/text forms of care-taking; involved in health care policies social-political decisions and engaged in social legislation matters
Self-Help Group for Women after Cancer	3	Yes/Yes/Yes	1976	Board of directors (4-7 members); patients	Ca. 12.000	To offer self-help groups and consultancy; organizing events and expert conferences; engaged in health care and social-political lobby work as interest representatives (e.g. in patients forum of German Medical Association); cooperation with other organizations and clinics
GBA	3	No/No/Y es	2004	Public legal entity four leading umbrella organizations of the self- governing German healthcare system.		To specify the concrete services to which patients and persons are insured; to issue directives for the benefit catalogue of the statutory health insurance funds (e.g. Disease management programs for the chronically ill); to conduct hearing procedures and consults external experts; to request expert opinions from independent scientific institutions; responsible for quality assurance of medical care in clinics and doctor’s practices.

**Table 2:** Overview of interviews: Affectedness, defended representation model and type of trust

PO	Interview	Affected	Representation model	Type of trust
1	1	n	both	both
	2	n	identity/trustee	vertical
2	1	y	delegate/identity	horizontal
	2	y	identity/trustee	horizontal
3	1	y	delegate/identity	horizontal
	2	n	trustee	vertical
	3	n	trustee	vertical
4	1	y	delegate	horizontal
	2	y	identity/trustee	horizontal
	3	y	delegate	both
FJC	FJC1	n	trustee	both, emphasis on vertical
	FJC2	n	trustee	both, emphasis on vertical
	FJC3	y	trustee	both, emphasis on vertical

Germany provides an excellent setting for studying the representation of patients in health care policy because of its large variety of organizations for chronic and rare diseases on the regional and national level [38]. Moreover, many formal structures are in place to involve patients in health policy making [39]. In 2003, the Law on Modernization of the Health Insurance has legislated patient involvement in the FJC, the highest board for decisions regarding the coverage medication or provisions by the public health care system.^2^ A more recent Bill of Patients’ Rights seeks to strengthen POs as participants in regional and national committees of health care policies [40].

To cover a broad spectrum of diseases in order to capture the variety of organizations and patients, we selected representatives of the German Society for Muscle Disease, German Alzheimer Society, Association for People with Locked-In-Syndrome, and the Self-Help Group for Women after Cancer. All POs are associations (*eingetragene Vereine, e.V.*), which requires them by law to have internal democratic structures such as voting, reporting, and participation [41]. Furthermore, we aimed to include a variety of types of organizations differing in their representation focus (umbrella organization, single disease) and representatives who were active volunteers or professional employees (Table 1).

All interviews were conducted in German (quotes anonymized and translated by HG and KJ) and were structured in the following way after the topic of the study was briefly introduced: the role and understanding of trust in general and within their organization, after which internal structures for decision-making within the PO were discussed, and finally they were asked to describe their own role. All interviews were tape-recorded and transcribed verbatim. For this study a content analysis [42], in which inductive and deductive analysis is combined, was undertaken by using a contestant comparative method: data were systematically reviewed for supportive and conflicting evidence for emergent themes and codes. We started inductively with an a priori coding list, based on the theoretical background (main codes: horizontal trust, vertical trust, delegate-representation and trustee-representation). A sample of three interviews was discussed between the authors to ensure agreement about the codes; all interviews have been cross-coded individually by HG and KJ. Note that we have coded separately for type of trust and representation style, in order to test our theoretical model in our empirical material. Additional codes and themes emerged from the data set (identity representation, being affected, challenges).

## Results

In the following, we will analyze the self-perception of representatives of POs and how they describe or claim their representation style and the role of trust, illustrated by quotes derived from the empirical material. Both representation styles and trust models were found in our material, delegate representation was related to horizontal trust, trustee representation was related to vertical trust.

### Representation in relation to trust

Regarding representation, both the trustee and the delegate style were defended by our interviewees. Those interviewees arguing in line with the idea of delegate representation (*N*=5), were often volunteers and/or affected and expressed the idea that discussion between the members should form the input for the representative’s standpoint. The members should find a common standpoint by discussing experiences and opinions, and bring that forward via a representative.

*And then they work it out, discuss a topic during several meetings and propose it to the representatives. The resulting statement is submitted to the representatives, who then again consider it and vote, and bring it back to the members. (Interview 1.1).*

These representatives have a more or less ‘deferential’ role towards their members; what they represent is not necessarily their own opinion; the representative should present what the group has decided.

*We represent a particular opinion, direction, goal. The ones, who represents us in the FJC or wherever, co-represents us; it is not about my personal opinion but about the opinion of the organization (Interview 4.1).*

Typically, the need for building trust *within* the group of patients was stressed for this type of representation. All representatives who argued in line with the delegate model, referred to the need for horizontal trust (Table 2). Five interviewees claimed horizontal trust as the most important trust type and five interviewees mentioned both types of trust as important and practiced (by them) in the organization (Table 2). This type of horizontal trust refers to situations where vulnerability plays a role, therefore discretion is considered an important element of trust. This type of trust is also based on having the same types of experiences, which results in mutual understanding and acceptance.

*By knowing each other well, we know, we can express our problems openly in the group and we know all the other ones have experienced or witnessed that in a similar way. (Interview 4.3).*

*I think that due to the mere fact that the affected individuals learn more about the disease [and] are somehow more self-confident, the trust within the group is increased (Interview 2.2).*

Some argued that it is not possible to have the support of all members for the decisions made, but including the members is nevertheless of utmost importance for developing organizational standpoints. The members must have had the chance to share their perspectives, even if not all are involved in, or agree with the final decision. In case of conflicting standpoints, some argued that it is important to take deviant opinions into account and document and communicate about them transparently. This type of decision-making gets challenging if there are more members within the organization, because such discussions cost much time and become ‘infinite’.

*The seven that had the time and interest to be involved […] we met in a small workshop, worked out a statement and the keywords and then I have summarized, so to speak, sent them back, they could then again give feedback and this is how we have organized the process. Not everyone participated in this, because it also has to do with resources, but each of them would have had the chance, so to speak, to get involved. (Interview 3.2).*

*There is constant exchange. So delegates are involved in an information flow. With all members, it is a little more difficult. It becomes very, you know the problems of referendums that then evolve to be endless. (Interview 1.1).*

The representatives note that not all members can and want to be part of the decision-making process. This can be due to physical or mental disability that makes travelling, participation or speaking difficult.

*[…] and then the affected ones indeed prefer to experience something together and the urge to become somehow involved and then, above all, transregional, nationwide, with long travelling, which is then, it is difficult in some cases we have some people who are also physically very fit, but they have problems to express themselves on the other side and for them it is difficult to follow those discussion processes (Interview 3.2).*

Other representatives argued in line with the trustee style (*n*=9), those were often professional spokespersons (Table 2). Trustee-representatives were argued to have certain skills such as knowledge about the political field.

*It is about personal competence, it would of course not be possible to represent the claims of others in such a* [political] *body, when one does not know how such a body functions at all (Interview 1.2).*

Typically, these interviewees argued that they should be credited trust in order to do a good job. This vertical trust is based upon the idea that the representatives can fulfill the expectations of the members and can decide in their interest. Most interviewees referring to the trustee model of representation (*N*=6), mentioned characteristics of vertical trust, some also mentioned some horizontal characteristics aside the vertical trust (Table 2). Professional competence was an important factor for trusting them.

*When I believe that this person also has certain skills, then I can also ask him in certain areas for advice and trust him. (Interview 1.2).*

Trust supports the representative autonomy for decision-making and is a precondition for the representative’s leeway to decide for the group. Transparency, regarding what is done in this space for autonomous decision-making, was mentioned to be crucial for maintaining trust in the representative. The decisions that are autonomously made by the representative should honestly and transparently be communicated back to the members, as this contributes to the reliability and trustworthiness of the representative.

*In this respect it is very, very important that there is a good relationship of trust here, which is in principle the basis for the work and this, this leads naturally to the fact that one has a certain autonomy to make decisions. (Interview 3.3).*

An exception to the binary model of representation and trust, delegate/horizontal and trustee/vertical, are representatives who stress the shared characteristics of ‘being affected’. These representatives stressed that inner-group trust may be fostered when the representative is also affected. Representation by an affected person was by some interviewees considered the most desirable form of representation, because being affected implies certain know-how and loyalty to the group of patients.

*Affectedness is our benchmark. Everything we do, and our work, our advisory work as well, is done basically from our experienced know-how and not by our professional skills, which we all have as well. We act based on our affectedness. (Interview 4.3).*

These representatives referring to the importance of the affectedness of the representatives can be described as ‘identity representatives’ because they stress the identical health status between representatives and the constituency. This means that identity representatives are ill themselves, and represent the patient-group suffering from the same condition as themselves. These representatives form an exception to the binary delegate/horizontal – trustee/vertical model, as the representation style refers to characteristics of both representation styles and both types of trust (Table 2). According to one interviewee *only* individuals affected by the disease can adequately represent the patients’ political interests and claims:

*[representation] is possible, if the person is directly affected, because they then carry this opinion in themselves, but it is not possible if I have ten people here who have an opinion and then go to the eleventh person and say, “Go and represent us over there”, that is not representation to me. (Interview 2.1).*

This representative also stated *that he even carries the members’ opinion in his body* when it comes to decision making. It is nearly an automatic process for him knowing what is right because of his own condition (notably he also used ‘somatic’ vocabulary to explain his view):

*Through this constant exchange and on top of that I'm even part of this body, so therefore I could never make any decisions taken by the head in conflict with the body, that's unthinkable for me. This does not apply to large organizations, because there is, the head is really - head and body are entirely separate. (Interview 2.1, emphasis by the authors).*

Furthermore an exceptional group are the representatives of the Federal Joint Committee (FJC) special representatives as they are assigned to act in this rather institutionalized decision-making body, and represent a variety of patient collectives. These representatives argued that FJC representatives are not ‘average’ patients but a sort of elite group, as they are able to act in the political arena, and have the competences to act in the interest of the patient collective.

*[…] the patient representatives of the FJC, are indeed a sort of elite, yes. They're not at all representative. Luckily. They are not representative, but they represent the interests of a large group. (Interview FJC3).*

Furthermore, these representatives stressed the need for distinguishing between individual and collective needs, a competence that is for individual members sometimes too hard. This may result in dissatisfaction with the representatives’ role and work.

*Complaints like “what do you actually do there? You do not represent me” hardly ever occur, of course it occurs in individual cases “I have written you, why is it not yet taken care of?!” But that, is not nearly as much as what, what you receive during the communication with group and self-help representatives as, as feedback, the things that are their concerns and then you carry it forward. (Interview FJC2).*

The representatives of the FJC stressed that the reputation and integrity of representatives are influential for perceiving them as trustworthy. The reputation of the person or of the organization they work for is important in granting the representative leeway and trusting them to make good decisions.

*Trust in individuals then is founded mainly on trusting the organization. Because people do not know indeed [name of the representative] but the people know in the best case the [Organization]. And then think ‘they are serious, they are not pharma infiltrated and they have been doing this for 30 years, and strive to improve and are also active on various subjects and somehow so. And when they send this person, then it will be ok. (Interview FJC3).*

Interestingly, the representatives of the FJC took a mediated stance rather between horizontal and vertical trust, as they argued simultaneously for the importance of group discussion, and being available for questions and concerns as well as for taking responsibility for the decisions made. They have argued that transparency and feedback of how decisions are made is required for being a good representative.

*It always has to do with how available you are, that means, I expose myself as self-critical, therefore I am available, I am there, I make it clear that I have this role, I, so far this has been an important prerequisite for trust to be addressed, but important is what we have decided and we stick to that, we have to share that information. (Interview FJC2).*

### Challenges for maintaining trust in representation relationships

Our interviewees also addressed several challenges and limitations for representation and trust-relationships. The underlying structure of communication is said to be crucial to avoid harming individual (private and political) autonomy because interests need to be articulated in a clear and imputable manner.

Trusting representatives partly limits one’s autonomy in terms in self-determination, by allowing the representative to make decisions for you. It was argued that as long as this delegation is based on trust, it allows the member at the same time to keep or extend his autonomy into the political sphere.

*It is a bit difficult, so it seems to me, concerning trust, that I place it in someone or in an institution, a part of my autonomy is lost, too. But this does not have to be the case, I think that I can maintain my autonomy and still give away, give away the representation in some things (Interview 1.1).*

Trust was mentioned by one interviewee to be especially challenging when ‘external’, or a non-affected person represent a collective, who lack sufficient knowledge about the wishes of the collective and about the condition.

*External people can [represent us]: If the required knowledge is there. Otherwise, they are a threat. (Interview 3.1).*

Collective representation is challenging as not all members are actively involved or politically interested. Therefore, several limitations were mentioned regarding the possibilities to include members in decision-making in general: many of the members are not interested in political decision-making for example:

*There are a few that take care of it and many who are not interested in it, and then there are elections and votes and delegates and there is a very small percentage of members who actually participate in these processes and are interested in the topics and a very large percentage who rather do not really care. (Interview 1.2).*

Political mobilization of citizens, passive as well as active members of associations or parties often pose a challenge for democratic self-organization in civil societies [43]. For POs there is an additional complicating factor: whether patients participate in the actions of the associations highly depends on the individual’s health condition. That means that being affected may set limitations for participation or getting into a representative role. Some interviewees mention that therefore POs need to have a healthy political representative, even if the patients might have special knowledge about the condition.

*I mean, someone cannot come to the one place, the other one cannot be at the other. The condition is different every day. So some cannot come today, because they are not in a good [physical] state and tomorrow another is not able to. (Interview 2.2).*

Representing an umbrella organization, with a constituency varying in wishes and needs, was considered a challenge for representation processes in general.

*So* **the** *patient does not exist, but there are very, there are different ways in health care, between acute and chronically ill, but also within the chronically ill there are differences, I would say, it is the case that we succeeded as far as possible regardless of the differences of interests, internally as well, to balance out as far as possible and then to come to a common standing. (Interview FJC1).*

Furthermore, it was stressed by the FJC representatives that a representative has to be independent, and has to prevent conflict of interests with the pharmaceutical industry, as that would harm the trust in the representative as a trustee for patients’ interests.

*There have been situations in which you ask the one person or the other, because there is a need for clarification, meaning how independent they are, and then there is certainly the possibility to dig deeper and ask and for example, concerning financial conflict of interests or so. (Interview FJC1).*

To summarize our empirical findings, we have found a binary model for representation and trust: vertical trust is required for trustee representation and horizontal trust is linked to delegate representation respectively. Our material has shown that different representatives of the same PO describe different representation styles and trust models (Table 2). Exceptions were the FJC representatives, who all defended a rather trustee-vertical model. Also hybrid combinations were found in the empirical material, mainly because of another type of representation: identity-representation, in which the shared identity of ‘being-affected’ is leading for representation and provides the basis for trust.

## Discussion

Bringing together conceptual considerations and empirical findings, we have seen that both types of trust are relevant for representatives of POs. Our theoretical distinctions are useful heuristics, while our empirical analysis illustrates the complexity of such concepts in practice. Our explorative study may not be representative for all POs in Germany, or for other countries, but has indicated that the plural and complex intermediary structure of patient organizations should not be underestimated. It is certainly recommendable to gain further insights into the heterogeneous roles and politics of and within POs. In the following, we will discuss our sample and findings using the insights of representation theory in order to shed light on the particularities of patient representatives of POs in comparison to other political actors. Secondly, we discuss the ‘identity representation’ that forms an exception to our findings, as they are not typically delegate/horizontal nor typical trustee/vertical oriented. Third, we will discuss the use of our study for other actors in the health care system and health care provision.

All interviewed representatives who described their role as trustee, mention vertical trust as important for their work – the process of trust building and deliberation in advance is subordinate to occasionally communicating certain decisions and planned advancements to the constituency. Furthermore, the trustee style together with its necessary resource of vertical trust seems to be linked to the professional status or background of the representatives: Most of the trustee representatives are employed at the association or are social workers, which refers back to the professionalization step that many POs have taken. These findings meet general assumptions about the nature of organizational structures in modern societies: Max Weber described professionalization of the roles of politicians to be a defining feature and result of bureaucratized and professionalized social systems [44]. Well-established elite theory states that the specialization and professionalization of political actors result from societal division of labor – while politicians and other professional officials need specific social and communicative competences in political or administrative contexts, lay people usually are not deemed to use these resources. The different capacities in turn legitimize the representational function of the specialized political and administrative personnel [45]. This is demonstrated by our empirical findings, where competence and reliability of the representative were argued to build the vertical trust for trustee representatives. The similarity of ‘traditional’ political structures in POs and the professionalization of POs and PO representatives, illustrates their growing role as serious actors in health policy making.

Patient representatives who worked as volunteers and were directly affected by the disease acted in nearly all cases as delegates and value horizontal trust. The delegate model benefits from intra-group trust. These shared values can be based on shared experiences, which again contribute to being seen as a trustworthy and reliable representative. Thus, delegate representation seems to benefit from ties of trust within the group and from a representative who is able to relate to the groups’ experiences. Horizontal trust and delegate representation seem to facilitate the collectivization from private issues and their transference into the political realm [5]: Relationships of trust are cultivated in safe spaces of self-help groups that help to address sensitive and even painful experiences. These experiences can subsequently be translated into shared political concerns by the representative in the relevant political forums. Research has pointed out that there are different approaches to include the patients’ view into health policy decision-making: One can implement inclusivity, deliberation and active citizenship by using citizens’ juries [41] or include them in scientific processes to gather insight into on patients’ perspectives [42] or evaluate online discussion forums [43]. Our interviews illustrate how POs aim to include their members and democratize their organizations in order to formulate the patients’ private concerns as political topics. The democratic structure we have seen in the POs we have included, may not be representative for POs in other settings, but is another indicator that POs are to be taken as serious as a CSO, as many other (health) political organizations.

The inclusion of affected people is an important presumption why POs are included in health care policy making. Interestingly, some statements from the interviews stressed that inclusion is enabled when representatives are affected themselves: Suffering from the same disease was referred to as a *sine qua non* for the representative to meet the patients’ political inclusion. This demand challenges the binary distinction between delegate and trustee representation because it doesn’t refer to the representatives’ behavior or attitudes but to their bodily features. We propose to interpret this constellation to understand as the demand for ‘identity representation’. The underlying claim is a relevant point for representation theory: Hannah F. Pitkin [22] referred to the concepts of ‘descriptive’ representation and ‘substantive’ representation. Substantive representation requires that representatives undertake the ‘right’ steps to advance the policy preferences of the constituency and serve their best interests. Descriptive representation refers to the representatives’ resemblance to their constituency – e. g. a woman representing a group of women [15, 23]. Our interview study indicates that this distinction may be relevant for the work of patient representatives. However, we cannot asses to what extent the representatives can be characterized as substantive representatives, because our data is limited to the self-perception of the representative and does not include the political outcome. The insistence on descriptive/identity representation made by the interviewee deserves some special attention. Descriptive representation strongly reflects ideas of identity politics. This concept was introduced to describe justifications for the necessity of self-representation by (historically) marginalized social groups. This kind of politics was actualized in the 1960s and 1970s as activism engaged in by status-based social movements organized around such categories as gender, race/ethnicity, sexuality, or other body-related issues like disability in contrast to class-based movements [44]. Even if the introduction of identity politics into the political discourse and practice helped to strengthen the political power of subaltern or stigmatized groups, one should not ignore the underlying epistemological suppositions: In her seminal work on identity, politics and intersectionality Kimberle Crenshaw summarized a problematic drawback which can be discussed as the pitfall of essentialism: “*The problem with identity politics [realized as descriptive/identity representation, the authors] is not that it fails to transcend differences, as some critics charge, but rather the opposite – that it frequently conflates or ignores intragroup differences*” [45], also [46]. As identity politics urges mobilization around a single axis of human traits (in our study: the illness as identity), it can pressure participants to classify that axis as their single defining feature, when in fact they may see themselves as complex individuals who cannot be represented so selectively or reductively. Identity representation could undermine the emancipatory idea of expanding the right of group representation and its pursuit for epistemic justice [46].

Patients with the same condition do not necessarily have the same opinions and interests by nature. Thus, negotiation among them, including the representative, constructs a forum to constitute a shared claim by which individual views can be transcended into political standpoints. Representatives enable this process by using a trustee or a delegate approach (or a combination of them), acknowledging that the represented patients themselves are not only private individuals, but shape the political constituency to which the representative is bound. Trust plays in that process of coming from individual wishes to collective and political standpoints a major role both in terms of horizontal as well as vertical trust.

The third point to discuss is the use of our analysis for other health care actors and health care provision. Representation takes place at several levels in the health care system. It is not unlikely that also on the micro- and meso-level, both representation styles are found, and both types of trust may be required. This includes the representation by proxy of incompetent patients. There are several standards to represent the incompetent patient’s wishes, such as the best interest standard and the substituted judgement standard (see e.g. [47]). Indeed, the best-interest model has similarities to the trustee model, as both allow the representative much leeway and is directed toward the ‘objective interests’, whereas the substituted judgement model has similarities to the delegate model, as both are based on the input and explicit wishes of the represented. Our study provides input to further study the role of trust within representative relations on the micro-level. Also on the meso-level patient interests are represented for example in hospital boards and research studies. These representatives may have relevant similarities to the practice of non-elected representatives as found within POs. Furthermore, a patient representative on the meso-level may face similar risks of equating one affected person with all affected people, which we have discussed as a risk of identity representation. Our study provides input to discuss such questions regarding representation and trust on these levels, but must be tested empirically.

## Conclusions

Our research concerns the ways in which trust and representation within POs are related. We have seen that most POs fit a binary distinction of delegate/horizontal and trustee/vertical, but identity representatives form an exception. POs have a complicated task of balancing between the inclusion of those affected and strategically aggregating a clear cut political claim. The two described representation styles, delegate and trustee, lean stronger on inclusion and deliberation, respectively political effectiveness and transparency.

As the constituency of a PO typically consist of a collective of affected people, it may be particularly difficult to mobilize or bring them together as a group, as the severity of the condition may hamper physical mobility or hinder those affected to become a representative themselves. Staying in contact with their constituency is therefore very important for representatives to legitimately represent a collective. The general need and advantage of including members in decision-making was well recognized in both trust–representation relationships: trust relations may be harmed by representatives who are not transparent about their own interests or when communication structures within the PO are opaque. Our empirical material has shown that both types of trust and both styles of representation are found within a single PO.

Trust plays in the process of coming from individual wishes to collective and political standpoints a major role both in terms of horizontal as well as vertical trust. Horizontal trust serves the communication between affected persons, and vertical trust allows representatives to decide. We therefore argue that both types of trust are required for the political and care related activities of POs. Beside our empirical findings one has to bear in mind that representation is not just a matter of responsibility towards or responsiveness of one representative to a group of specific, clearly defined members of the organization. In fact, representational acts always transcend the orientation towards a specific, organized group of members in a symbolic and practical way. Representation invokes the normative political claim of standing for a whole constituency of political subjects (who can benefit from the outcomes of the political action in principle). The concrete politics and representation processes in POs, which use trust as an operative catalyst, can be seen as the nucleus of activities to improve the lives of people being affected with diseases in a society.

## Footnotes

1 The focus of this paper is on the self-perception of the representatives, rather than on the perception of these representatives by other actors in the political arena.

2 “The Federal Joint Committee (G-BA) is a public legal entity comprising the four leading umbrella organizations of the self-governing German healthcare system: the National Associations of Statutory Health Insurance Physicians and Dentists, the German Hospital Federation, and the Central Federal Association of Health Insurance Funds. In addition to these four pillar organizations, patient representatives also participate in all sessions; they are entitled to put topics on the agenda, but not to vote.” [48].

3 Other papers have been published based on the primary analysis of this material, see: Beier K, Jordan I, Wiesemann C, Schicktanz S: Understanding collective agency in bioethics. Med Health Care Philos. 2016; doi:10.1007/s11019-016-9695-4. And Jordan, I, Schicktanz S: Kollektive Patientenautonomie: Theorie und Praxis eines neuen bioethischen Konzepts. Patientenautonomie: Theoretische Grundlagen - Praktische Anwendungen. Mentis:Münster, 2013.
